# Deep learning-based, fully automated, pediatric brain segmentation

**DOI:** 10.1038/s41598-024-54663-z

**Published:** 2024-02-22

**Authors:** Min-Jee Kim, EunPyeong Hong, Mi-Sun Yum, Yun-Jeong Lee, Jinyoung Kim, Tae-Sung Ko

**Affiliations:** 1 Department of Pediatrics, Asan Medical Center Children’s Hospital, Ulsan University College of Medicine, 88, Olympic-ro 43-Gil, Songpa-Gu, Seoul, 05505 South Korea; 2 VUNO Inc., Seoul, South Korea; 3 Department of Pediatrics, Kyungpook National University Hospital and School of Medicine, Kyungpook National University, Daegu, South Korea

**Keywords:** Dravet syndrome, Deep learning-based segmentation, Convolutional neural network, VUNO Med-DeepBrain, Neurodevelopmental disorders, Computational neuroscience, Image processing, Software

## Abstract

The purpose of this study was to demonstrate the performance of a fully automated, deep learning-based brain segmentation (DLS) method in healthy controls and in patients with neurodevelopmental disorders, *SCN1A* mutation, under eleven. The whole, cortical, and subcortical volumes of previously enrolled 21 participants, under 11 years of age, with a *SCN1A* mutation, and 42 healthy controls, were obtained using a DLS method, and compared to volumes measured by Freesurfer with manual correction. Additionally, the volumes which were calculated with the DLS method between the patients and the control group. The volumes of total brain gray and white matter using DLS method were consistent with that volume which were measured by Freesurfer with manual correction in healthy controls. Among 68 cortical parcellated volume analysis, the volumes of only 7 areas measured by DLS methods were significantly different from that measured by Freesurfer with manual correction, and the differences decreased with increasing age in the subgroup analysis. The subcortical volume measured by the DLS method was relatively smaller than that of the Freesurfer volume analysis. Further, the DLS method could perfectly detect the reduced volume identified by the Freesurfer software and manual correction in patients with *SCN1A* mutations, compared with healthy controls. In a pediatric population, this new, fully automated DLS method is compatible with the classic, volumetric analysis with Freesurfer software and manual correction, and it can also well detect brain morphological changes in children with a neurodevelopmental disorder.

## Introduction

Neurodevelopmental disorders (NDD) are highly prevalent among children aged 3 to 17 in the United States, affecting approximately 17% of this population^1^. Recent advancements in genetic technologies have led to the identification of genetic causes in 15–53% of NDD cases^2^, but comprehensive phenotypic evaluation, including laboratory tests and neuroimaging, remains crucial for precise genetic diagnosis. Eventually, these evaluations provide insights into the underlying mechanisms and potential biomarkers associated with these disorders.

Since the advent of high-resolution, three-dimensional (3D) structural magnetic resonance imaging (MRI) of the human brain, brain morphometric analysis has been widely applied in various neurodevelopmental diseases^3–6^ and the features, such as regional volume and thickness, have been developed as biomarkers of disease states or treatment responses^7,8^. Additionally, repeated MRI scans can typically be made on the same individual, due to their non-invasive and radiation-free characteristics, enabling the visualization of longitudinal changes in normal brain development and the distinguishing of atypical trajectories in pediatric patients with neurodevelopmental diseases^9,10^.

To conduct brain morphometric analysis effectively in both research and clinical settings, precise segmentation of T1-weighted brain MRI into anatomical regions is an essential component of quantitative analysis^11^. Given that manual delineation of structural parameters is both labor-intensive and prone to inter-rater variability, standardized and automated processing approaches such as mni_autoreg^12^, SPM^13^, Freesurfer^14,15^, and FSL^16,17^ are widely used to label novel target images in adults. Nevertheless, the pediatric brain is substantially distinct from its adult counterpart. Consequently, these templates, predominantly derived from adult data, may not be ideally suited for pediatric applications^11^.

Owing to these challenges, innovative methods are being incessantly developed to address the constraints inherent to existing template-based approaches. Recently, deep learning has emerged as a promising methodology for precise brain segmentation^18^. Deep learning encompasses neural networks exceeding five layers, which facilitate the extraction of hierarchical features directly from raw images. Given their capability for autonomous learning, these networks demonstrate remarkable outcomes and broad generalizability when trained on extensive datasets^11,18–20^. In the field of neonate and infant brain segmentation, including whole brains or lesions, convolutional neuronal networks (CNNs) are the most commonly used^11^. In this context, a state-of-the-art deep-learning based segmentation (DLS) methodology, named VUNO Med-Deep Brain, has been introduced in adult cohorts with Alzheimer’s disease^21^.

In a previous study conducted by our team, we utilized the Freesurfer software coupled with manual adjustments to execute brain morphometric analyses. This yielded the first comprehensive findings on developmental brain alterations and volumetric disparities in regional structures among epilepsy patients harboring an *SCN1A* gene mutation^22^. The *SCN1A* gene (MIM#182389), responsible for encoding the alpha 1 subunit of the voltage-gated sodium channel, has mutations that can lead to an array of neurodevelopmental disorders, including epilepsy^23^.

To ascertain the efficacy of the newly devised deep-learning based morphometric analysis for pediatric cohorts, we compared the structural parameters derived from the DLS method for healthy children aged under 11 with those obtained from the Freesurfer software supplemented by manual corrections. For a more granular assessment of the age-specific accuracy of the DLS approach, we performed subgroup analyses focusing on three distinct age groups: under two years, between two to six years, and older than six years. Furthermore, to gauge the robustness of the DLS technique, we juxtaposed regional volumes between pediatric patients (below 11 years) with an *SCN1A* mutation and their healthy counterparts.

## Results

### Baseline demographics of patients and control group

Twenty-one patients with a *SCN1A* mutation and 42 healthy controls, previously selected and analyzed by our center, were re-analyzed in this study (age range; 2.0–10.5). To delineate the age-specific performance of the DLS method, we classified the control group into three different age subgroups: age ≤ 2 years (n = 12, 28.6%), 2 < Age ≤ 6 years (n = 18, 42.9%), and 6 < Age ≤ 10 years (n = 12, 28.6%). The baseline demographics are shown in Table 1 of a previous study^22^.

**Table 1:** Whole brain volume analysis between two methods, DLS and Freesurfer with manual correction methods in the control group.

Parcellation region	Overall (n = 42, 100%)			Age ≤ 2 (n = 12, 28.6%)			2 < Age ≤ 6 (n = 18, 42.9%)			Age > 6 (n = 12, 28.6%)		
	DLS, mean	Free-surfer, mean	p-value	DLS, mean	Free-surfer, mean	p-value	DLS, mean	Free-surfer, mean	p-value	DLS, mean	Free-surfer, mean	p-value
Total brain	1127.1	1073.4	0.014	1076.8	1036.3	0.123	1120.4	1044.8	0.030	1187.3	1153.3	0.848
Total GM	610.4	701.8	0.239	606.9	695.0	0.127	612.8	685.9	0.141	610.2	732.6	0.019
Cortical GM	566.3	547.6	0.006	565.0	545.4	0.012	569.1	540.2	0.135	563.5	560.8	1
Subcortical GM	44.1	55.9	** < 0.001**	41.9	53.3	** < 0.001**	43.7	54.0	** < 0.001**	46.7	61.5	** < 0.001**
Total WM	358.9	348.2	0.025	323.9	321.7	1	354.2	335.8	0.086	400.9	393.3	0.35
Total cerebellum	107.3	98.8	** < 0.001**	101.5	94.7	0.004	103.6	93.1	0.014	118.6	111.6	0.737
Cerebellar WM	21.2	23.9	** < 0.001**	17.7	20.2	0.019	20.6	23.6	0.014	25.7	28.1	0.28
Cerebellar GM	128.5	122.8	** < 0.001**	119.2	114.9	** < 0.001**	124.2	116.7	** < 0.001**	144.3	139.7	** < 0.001**
Total ventricle	13.3	14.1	0.01	12.7	13.0	1	13.6	14.2	0.039	13.7	14.9	0.135

Volume were normalized by ICV and expressed in mL.

The paired *t* test was applied.

Bold font indicates statistical significance (*p* < *0.001*).

*GM* gray matter, *WM* white matter.

### Brain volume analysis in control group measured by two different methods

#### Whole-brain volume analysis

**Figure 1:**
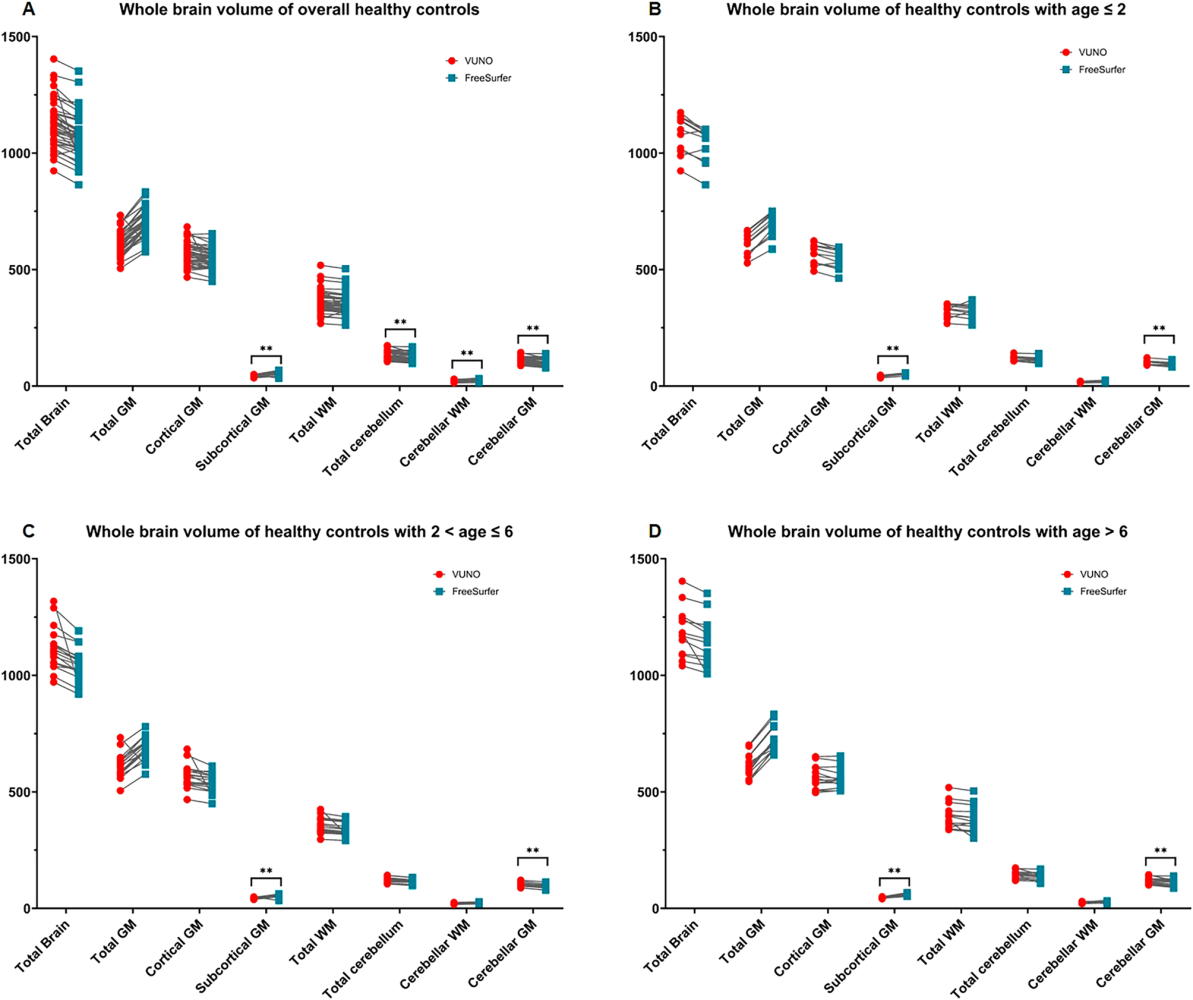
Whole brain volume analysis in a 42 healthy control by the DLS method and Freesurfer with manual correction. (**A**) Whole brain volume of overall healthy controls (**B**) Whole brain volume of healthy controls with age ≤ 2 years (**C**) Whole brain volume of healthy controls with 2 < age ≤ 6 (**D**) Whole brain volume of healthy controls with age > 6 The blue dots showed the volume measured by Freesurfer with manual correction; The red dots showed the volume measured by the DLS method. **p-values < 0.001.

The volumes of total brain, total gray matter, cortical gray matter, and total white matter measured by DLS method were not different from those measured by Freesurfer with manual correction. The volume of subcortical gray matter measured by DLS was significantly smaller, and the total cerebellum measured by DLS was larger than that measured by Freesurfer with manual correction methods. (Table 1 and Fig. 1).

After subgroup analysis according to age, the volume differences of the subcortical and cerebellar gray matter between the two methods were revealed to be consistent in all three subgroups.

#### Cortical parcellated volume analysis

Among the 68-parcellated areas measured by the two methods, volume differences were found in only 7 areas (Table 2). The volumes of the right caudal middle frontal, right frontal pole, left inferior parietal, left fusiform, right lateral occipital, and right insular cortex were significantly smaller. However, the left parahippocampus volume was significantly larger when measured by DLS, than when measured by Freesurfer with manual correction methods. Subgroup analysis between the two methods exhibited a significant volume difference in the right insular cortex among the ≤ 2 years of age group.

**Table 2:** Cortical volume analysis between the two methods, DLS and Freesurfer with manual correction methods in control group.

Parcellation region	Overall (n = 42, 100%)			Age ≤ 2 years (n = 12, 28.6%)			2 < Age ≤ 6 years (n = 18, 42.9%)			Age > 6 years (n = 12, 28.6%)		
	DLS, Mean	Free-surfer, mean	p-value	DLS, mean	Free-surfer, mean	p-value	DLS, mean	Free -surfer, mean	p-value	DLS, mean	Free-surfer, mean	p-value
Frontal												
Superior frontal gyrus, left	25.6	25.6	1	24.9	25.0	1	25.7	25.6	1	26.0	26.2	1
Superior frontal gyrus, right	24.2	24.5	1	23.4	24.0	1	24.6	24.6	1	24.3	24.9	1
Rostral middle frontal, left	18.8	19.0	1	18.6	19.1	1	18.8	18.5	1	18.8	19.8	1
Rostral middle frontal, right	19.4	20.0	1	18.7	20.1	1	19.8	19.7	1	19.6	20.4	1
Caudal middle frontal, left	7.2	6.9	1	6.8	6.7	1	7.4	7.0	1	7.4	7.1	1
Caudal middle frontal, right	7.5	6.5	** < 0.001**	7.3	5.9	0.552	8.0	6.7	0.136	7.1	6.8	1
Pars opercularis, left	5.5	5.5	1	4.9	4.9	1	5.8	5.6	1	5.8	5.9	1
Pars opercularis, right	4.6	4.7	1	4.4	4.3	1	4.8	4.9	1	4.6	4.9	1
Pars triangularis, left	4.6	4.5	1	4.7	4.3	1	4.6	4.5	1	4.5	4.6	1
Pars triangularis, right	5.3	5.2	1	5.3	5.3	1	5.2	5.1	1	5.5	5.4	1
Pars orbitalis, left	2.8	2.6	1	2.6	2.6	1	2.8	2.6	1	2.9	2.6	1
Pars orbitalis, right	3.0	3.2	1	2.9	3.2	1	2.9	3.1	1	3.1	3.3	1
Lateral orbitofrontal, left	9.2	8.5	0.014	9.1	8.5	1	9.3	8.3	1	9.3	8.9	1
Lateral orbitofrontal, right	8.1	8.1	1	8.0	8.2	1	8.2	7.9	1	8.1	8.3	1
Medial orbitofrontal, left	6.2	5.8	1	6.4	6.2	1	6.1	5.6	1	6.2	5.7	1
Medial orbitofrontal, right	6.4	6.1	1	6.6	6.3	1	6.3	6.1	1	6.3	6.0	1
Precentral, left	14.5	14.9	1	13.9	14.4	1	14.7	14.8	1	14.7	15.4	1
Precentral, right	14.4	14.6	1	13.9	13.7	1	14.5	14.9	1	14.7	15.2	1
Paracentral, left	4.1	4.2	1	3.9	4.0	1	4.2	4.3	1	4.3	4.5	1
Paracentral, right	4.1	4.7	0.044	4.0	4.4	1	4.2	4.7	1	4.3	4.9	1
Frontal pole, left	1.4	1.1	0.002	1.6	1.3	1	1.4	1.2	0.524	1.1	1.0	1
Frontal pole, right	1.9	1.5	** < 0.001**	2.2	1.6	0.033	1.9	1.5	1	1.7	1.3	1
Rostral anterior cingulate, left	2.9	3.0	1	2.9	3.1	1	3.0	3.0	1	2.9	2.9	1
Rostral anterior cingulate, right	2.2	2.3	1	2.1	2.3	1	2.2	2.2	1	2.3	2.5	1
Caudal anterior cingulate, left	1.8	2.0	1	1.7	2.0	1	2.0	1.9	1	1.7	2.0	0.218
Caudal anterior cingulate, right	2.3	2.6	1	2.1	2.5	1	2.4	2.5	1	2.4	2.8	1
Parietal												
Superior parietal, left	17.9	16.8	1	18.1	16.9	1	17.9	16.9	1	17.5	16.4	1
Superior parietal, right	16.6	16.3	1	17.0	16.3	1	16.5	16.4	1	16.3	16.0	1
Inferior parietal, left	16.3	17.0	1	17.4	17.2	1	15.2	16.3	1	16.9	17.8	1
Inferior parietal, right	21.8	19.4	** < 0.001**	21.7	19.6	0.128	22.1	18.8	0.235	21.7	20.2	1
Supramarginal, left	14.4	13.6	1	13.7	13.4	1	15.0	13.7	1	14.3	13.7	1
Supramarginal, right	11.6	12.8	0.004	11.0	12.9	1	11.7	12.7	1	12.1	12.9	1
Postcentral, left	11.4	12.1	0.099	11.6	12.3	1	11.1	11.8	1	11.8	12.3	1
Postcentral, right	11.1	11.9	0.256	11.3	12.2	1	10.9	11.7	1	11.3	12.0	1
Precuneus, left	12.6	12.8	1	13.0	13.0	1	12.6	12.9	1	12.0	12.5	1
Precuneus, right	13.4	12.9	1	14.2	13.2	1	13.4	12.9	1	12.7	12.5	1
Posterior cingulate, left	3.9	3.9	1	3.9	3.9	1	3.9	3.8	1	3.8	3.9	1
Posterior cingulate, right	4.0	4.0	1	3.8	3.7	1	4.1	4.1	1	4.1	4.2	1
Isthmus cingulate, left	3.4	3.3	1	3.6	3.4	1	3.4	3.4	1	3.1	3.2	1
Isthmus cingulate, right	3.3	3.2	1	3.6	3.4	1	3.3	3.2	1	3.1	3.0	1
Temporal												
Superior temporal, left	14.1	13.2	1	13.8	12.8	1	13.9	13.0	1	14.5	13.8	1
Superior temporal, right	13.3	13.3	1	13.2	12.7	1	13.6	13.5	1	13.0	13.6	1
Middle temporal, left	12.4	12.2	1	12.0	12.0	1	12.4	11.6	1	12.8	13.1	1
Middle temporal, right	15.3	13.7	0.003	15.1	13.5	1	14.9	13.1	1	15.9	14.9	1
Inferior temporal, left	12.8	11.2	0.014	12.7	10.8	0.066	13.1	10.6	0.408	12.5	12.5	1
Inferior temporal, right	11.2	10.4	1	11.3	10.6	1	11.1	9.5	1	11.2	11.7	1
Banks of the sts, left	3.0	2.8	1	2.9	2.9	1	3.1	2.8	1	3.1	2.9	1
Banks of the sts, right	2.8	2.8	1	2.6	2.5	1	2.9	2.8	1	2.9	3.0	1
Fusiform, left	12.4	11.1	** < 0.001**	12.5	10.4	0.079	12.6	11.0	0.323	11.8	11.7	1
Fusiform, right	11.0	10.4	1	10.9	10.3	1	11.4	10.3	1	10.5	10.7	1
Transverse temporal, left	1.5	1.5	1	1.5	1.5	1	1.6	1.5	1	1.6	1.4	1
Transverse temporal, right	1.4	1.2	0.002	1.3	1.2	1	1.4	1.2	1	1.4	1.1	0.069
Entorhinal, left	1.8	1.7	1	1.8	1.5	1	1.8	1.7	1	1.9	1.9	1
Entorhinal, right	1.5	1.5	1	1.5	1.5	1	1.3	1.4	1	1.8	1.6	1
Temporal pole, left	2.6	2.6	1	2.6	2.6	1	2.7	2.6	1	2.4	2.8	1
Temporal pole, right	2.4	2.3	1	2.3	2.5	1	2.3	2.1	1	2.6	2.3	1
Parahippocampal, left	2.0	2.4	** < 0.001**	1.9	2.2	1	2.0	2.4	0.034	2.2	2.5	1
Parahippocampal, right	2.0	2.1	1	2.0	1.9	1	2.0	2.0	1	2.2	2.3	1
Occipital												
Lateral occipital, left	14.6	13.1	0.004	15.1	13.2	1	14.9	12.6	0.008	13.6	13.6	1
Lateral occipital, right	15.4	13.1	** < 0.001**	16.9	13.4	0.003	15.1	12.8	1	14.5	13.2	1
Lingual, left	7.9	8.0	1	8.5	8.3	1	8.1	8.1	1	7.0	7.4	1
Lingual, right	8.0	7.9	1	8.3	8.2	1	8.4	8.0	1	7.2	7.5	1
Cuneus, left	3.4	3.7	1	3.6	4.0	1	3.5	3.8	1	3.3	3.4	1
Cuneus, right	3.8	4.0	1	4.2	4.3	1	3.7	4.0	1	3.7	3.8	1
Pericalcarine, left	2.5	2.5	1	2.7	2.7	1	2.6	2.5	1	2.4	2.3	1
Pericalcarine, right	2.6	2.8	1	2.7	2.9	1	2.6	2.8	1	2.5	2.5	1
Insular cortex, left	7.0	6.9	1	7.0	6.9	1	6.9	6.6	1	7.1	7.2	1
Insular cortex, right	7.2	6.9	** < 0.001**	7.2	6.8	** < 0.001**	7.1	6.5	0.601	7.4	7.4	1

Data are presented as differences between means.

Paired *t* test and Bonferroni correction was applied.

Bold font indicates statistical significance (*p* < *0.001*).

#### Subcortical volume analysis

**Figure 2:**
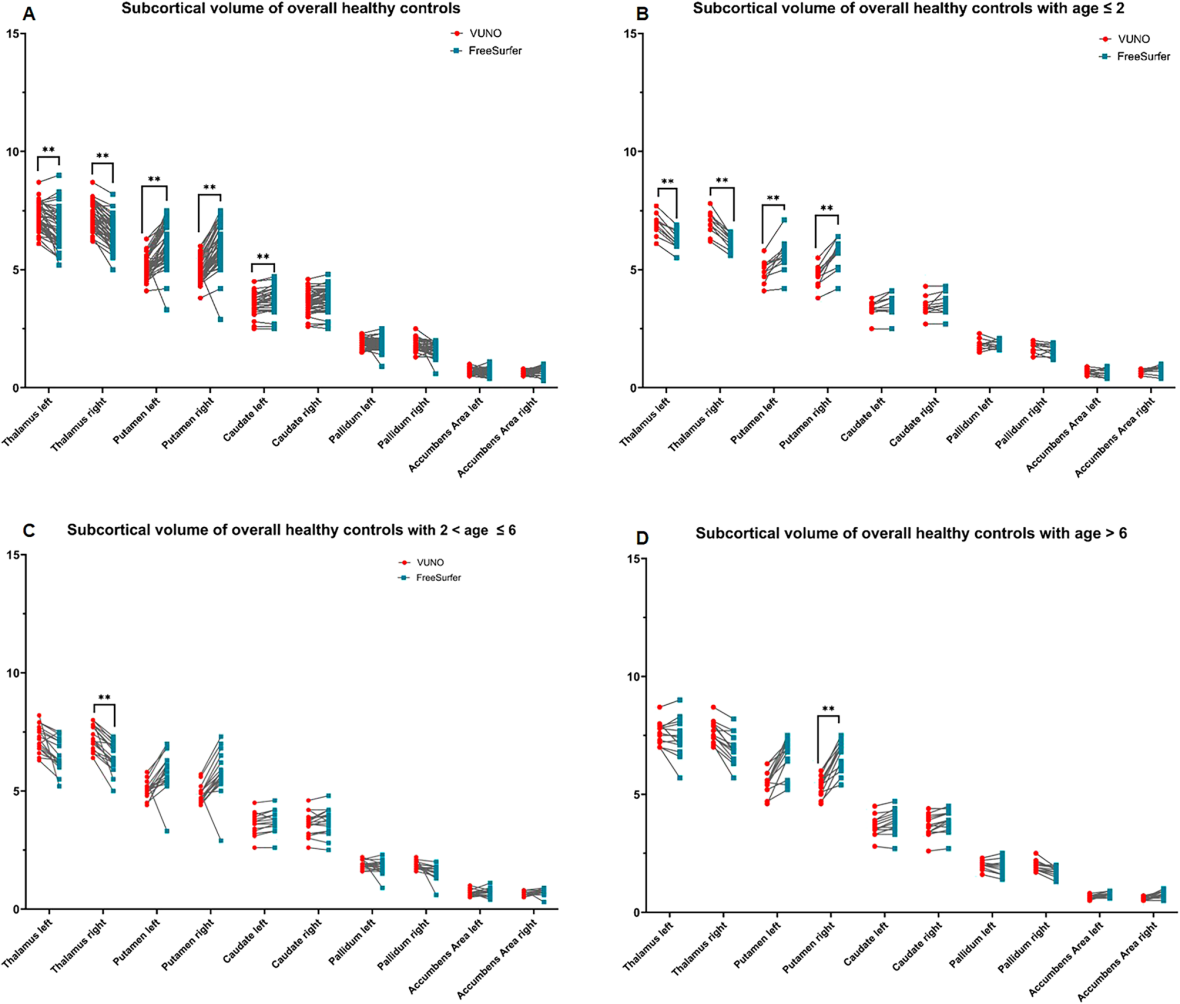
Subcortical volume analysis in a 42 healthy control by the DLS method and Freesurfer with manual correction. (**A**) Whole brain volume of overall healthy controls (**B**) Whole brain volume of healthy controls with age ≤ 2 years (**C**) Whole brain volume of healthy controls with 2 < age ≤ 6 (**D**) Whole brain volume of healthy controls with age > 6 The blue dots showed the volume measured by Freesurfer with manual correction; The red dots showed the volume measured by the DLS method. **p-values < 0.001.

The measured volume of both thalami, both putamen, and left caudate were significantly different between those measured by the DLS method and those by the Freesurfer with the manual correction method (Table 3 and Fig. 2). In the ≤ 2 years age group, both thalami were significantly larger and the right putamen was significantly smaller in the volume measured by the DLS method, compared to that by Freesurfer with the manual correction method. These differences were also observed in volumes of the right thalamus in the 2 < Age ≤ 6 years group and the right putamen in the 6 < Age ≤ 10 years group.

**Table 3:** Subcortical volume analysis between the two methods, DLS and Freesurfer with manual correction methods in control group.

Parcellation region	Overall (n = 42, 100%)			Age ≤ 2 years (n = 12, 28.6%)			2 < Age ≤ 6 years (n = 18, 42.9%)			Age > 6 years (n = 12, 28.6%)		
	DLS, mean	Free-surfer, mean	p-value	DLS, mean	Free-surfer, mean	p-value	DLS, mean	Free-surfer, mean	p-value	DLS, mean	Free-surfer, mean	p-value
Thalamus, left	7.2	6.7	** < 0.001**	6.9	6.3	** < 0.001**	7.2	6.5	0.047	7.6	7.6	1
Thalamus, right	7.3	6.5	** < 0.001**	7.0	6.2	** < 0.001**	7.3	6.4	** < 0.001**	7.7	7.1	0.002
Putamen, left	5.2	6.0	** < 0.001**	5.0	5.7	0.034	5.0	5.7	0.075	5.6	6.7	0.005
Putamen, right	5.0	6.0	** < 0.001**	4.8	5.8	** < 0.001**	4.8	5.7	0.012	5.4	6.7	** < 0.001**
Caudate, left	3.5	3.7	** < 0.001**	3.4	3.5	1	3.6	3.7	0.477	3.7	3.9	0.117
Caudate, right	3.6	3.7	0.012	3.4	3.6	1	3.6	3.7	1	3.7	3.9	0.021
Pallidum, left	1.9	1.8	1	1.8	1.9	1	1.9	1.8	1	2.0	1.9	1
Pallidum, right	1.9	1.6	0.002	1.7	1.6	1	1.8	1.6	0.492	2.0	1.8	0.013
Accumbens, left	0.7	0.7	1	0.7	0.7	1	0.7	0.7	1	0.7	0.7	1
Accumbens, right	0.6	0.7	0.002	0.7	0.7	1	0.6	0.7	0.782	0.6	0.8	0.123

Volume were normalized by ICV and expressed in mL.

Paired *t* test and Bonferroni correction was applied.

Bold font indicates statistical significance (*p* < *0.001*).

### Group comparison of each volume between patients with a SCN1A mutation and healthy control measured by DLS

#### Whole-brain volume analysis

**Figure 3:**
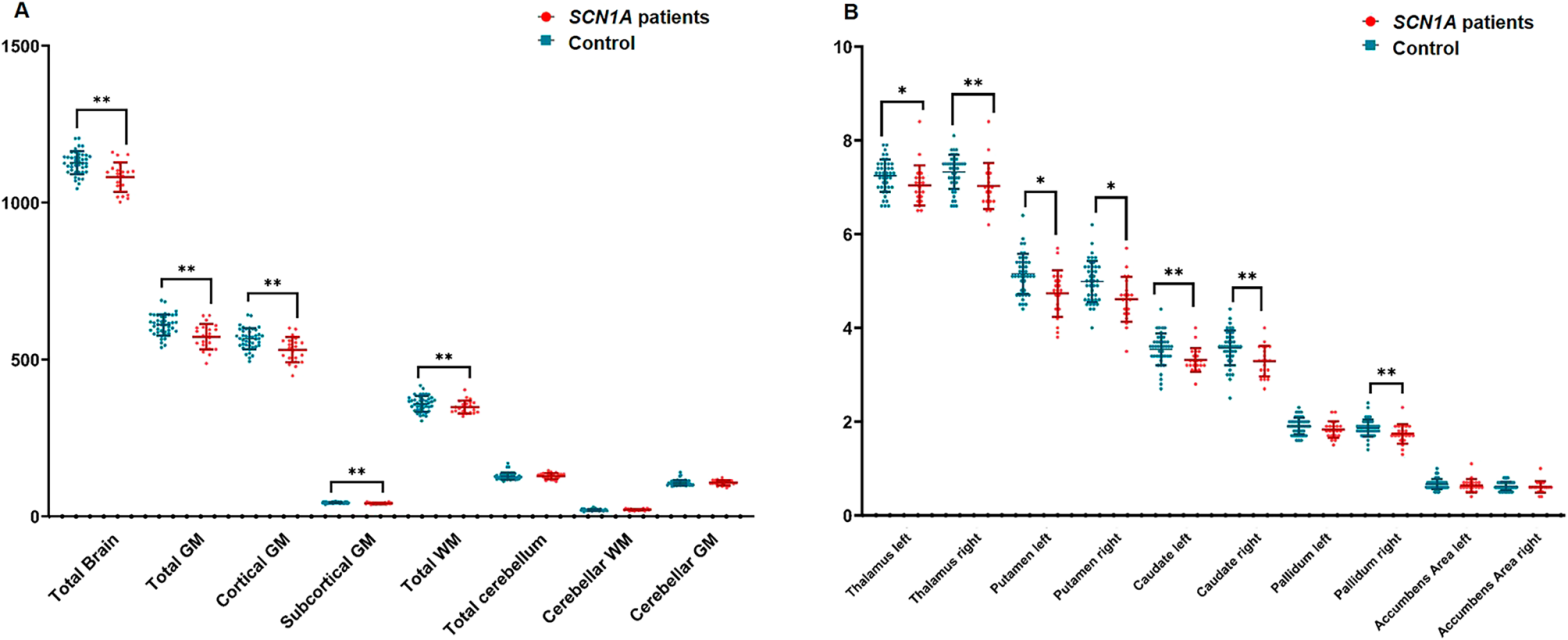
Group comparison of whole brain (**A**) and subcortical (**B**) parcellated volumes by deep-learning based segmentation (DLS) method. The blue dots showed the volumes of healthy controls; The red dots showed the volume of *SCN1A* patients **p-values < *0.001.*

After adjusting for the sex, age and ICV for each group, the volumes of the total brain, total gray matter, cortical gray matter, subcortical gray matter, and total white matter were found to be significantly smaller in the patient group than in the controls (Table 4 and Fig. 3A) as is consistent with a previous study^22^.

**Table 4:** Group comparison of whole brain, subcortical and cortical parcellated volumes by deep-learning based segmentation (DLS) method.

	Control mean (SD)	Patients mean (SD)	Difference between means (%)	p-value
Whole brain volume analysis				
Total brain	1127.07 (155.73)	1041.66 (228.22)	− 7.8	< 0.001**
Total GM	610.37 (6.878)	557.01 (11.17)	− 9.0	< 0.001**
Cortical GM	566.30 (6.53)	516.36 (10.65)	− 9.1	< 0.001**
Subcortical GM	44.07 (0.62)	40.66 (0.79)	− 8.0	< 0.001**
Total WM	358.87 (.8627.97)	331.67 (10.63)	− 7.8	< 0.001*
Total cerebellum	128.51 (2.59)	124.72 (3.28)	− 3.0	0.088*
Cerebellar WM	21.21 (0.76)	20.78 (0.87)	− 2.1	0.413*
Cerebellar GM	107.30 (1.90)	103.89 (2.50)	− 3.2	0.086

	Left, difference between means (%)	Left, p-value	Right, difference between means (%)	Right, p-value
Cortical volume analysis				
Frontal				
Superior frontal gyrus	− 8.4	0.015	− 9.5	0.024
Rostral middle frontal	− 10.3	0.045	− 12.2	0.016
Caudal middle frontal	− 4.5	1	− 9.5	1
Pars opercularis	− 13.2	0.154	− 8.0	1
Pars triangularis	− 10.9	1	− 9.1	1
Pars orbitalis	− 11.7	0.558	− 9.2	0.226
Lateral orbitofrontal	− 13.1	< 0.001**	− 11.9	** < 0.001**
Medial orbitofrontal	− 3.7	1	− 4.9	1
Precentral	− 7.6	< 0.001**	− 8.2	** < 0.001**
Paracentral	− 1.2	1	− 8.9	0.397
Frontal pole	− 2.9	1	− 10.3	0.807
Rostral anterior cingulate	− 18.2	0.032	− 15.4	0.179
Caudal anterior cingulate	− 8.9	1	− 5.7	1
Parietal				
Superior parietal	− 9.0	0.035	− 3.2	1
Inferior parietal	− 12.0	< 0.001**	− 18.0	** < 0.001**
Supramarginal	− 4.8	1	− 7.1	0.752
Postcentral	− 3.1	1	− 7.1	1**
Precuneus	− 10.7	0.169	− 11.8	0.062
Posterior cingulate	− 10.1	0.296	− 10.1	0.445
Isthmus cingulate	− 9.6	0.017	− 13.5	< 0.001**
Temporal				
Superior temporal	− 7.6	1	− 6.9	1
Middle temporal	− 11.8	0.368	− 14.0	0.001**
Inferior temporal	− 7.0	1	− 2.8	1
Banks of the STS	− 17.3	** < 0.001**	− 15.1	0.022
Fusiform	− 8.8	0.553	− 8.4	0.984
Transverse temporal	− 0.6	1	− 7.5	1
Entorhinal	− 11.7	0.959	− 2.4	1
Temporal pole	− 7.1	1	− 4.5	1
Parahippocampal	− 16.0	** < 0.001**	− 9.4	0.162
Occipital				
Lateral occipital	− 12.3	0.064	− 9.9	1
Lingual	− 6.1	1	− 8.3	1
Cuneus	− 7.3	1	− 8.5	1
Pericalcarine	− 6.7	1	− 6.1	1
Insular cortex	− 6.3	0.537	− 8.0	** < 0.001**
Subcortical volume analysis				
Thalamus	− 5.4	**0.006**	− 6.8	** < 0.001**
Putamen	− 10.2	0.003**	− 9.6	0.005**
Caudate	− 10.4	< 0.001**	− 12.5	< 0.001**
Pallidum	− 5.7	0.123	− 9.4	**0.001**
Accumbens	− 6.5	1**	− 4.4	1**

General linear models were used to account for correlation by matching of age and gender.

Bold font indicates statistical significance (*p* < 0.001) after Bonferroni correction.

Volume were normalized by ICV and expressed in mL.

*GM* gray matter, *WM* white matter, *SD* standard deviation.

**p-values < 0.001, and *p-values < *0.05*: the areas with significant differences between two group had been shown in previous study using FreeSurfer with manual correction method.

#### Cortical parcellated volume analysis

The 34 cortical parcellated regions per hemisphere between the two groups; healthy control and patients with *SCN1A* mutation were compared (Table 4 and Supplementary Table 1). In comparison to heathy controls, patients showed significantly decreased volumes in both the lateral orbitofrontal, precentral, and inferior parietal, right isthmus cingulate, right middle temporal, left Banks of the STS, left parahippocampal, and right insular cortex compared to healthy control.

#### Subcortical volume analysis

A subcortical volume analysis was performed to compare the volumes of the subcortical structures (thalamus, caudate, putamen, pallidum, accumbens area) between the patients and the controls (Table 4, Supplementary Table 1 and Fig. 3B). The patients showed significantly smaller volumes of both the thalami, putamen, and caudate, and right pallidum than those of healthy controls.

### Group comparison of each volume between patients with a SCN1A mutation and healthy control measured by DLS

In the assessment of our segmentation algorithm's efficacy, we computed the Dice Similarity coefficient (DSC), along with precision and recall metrics, across whole, cortical, and subcortical brain regions as delineated in Table 5. Our findings revealed cortical gray matter showed relatively low performance compared to other whole brain areas. Notably, specific areas such as the frontal pole, entorhinal cortex, temporal pole, and nucleus accumbens exhibited suboptimal performance, with performance values falling below 0.7, in both cortical and subcortical analyses.

**Table 5:** Mean dice score coefficient (DSC), precision, and recall values between two methods, DLS and Freesurfer with manual correction methods in control, SCN1A patients, and total patients.

	Dice coefficient			Precision			Recall		
	Total	Healthy control	SCN1A patients	Total	Healthy control	SCN1A patients	Total	Healthy control	SCN1A patients
Total brain	0.79	0.79	0.80	0.80	0.79	0.80	0.80	0.80	0.81
Total GM	0.80	0.80	0.80	0.81	0.80	0.81	0.80	0.80	0.81
Cortical GM	0.78	0.77	0.78	0.78	0.78	0.78	0.79	0.78	0.79
Subcortical GM	0.82	0.82	0.82	0.83	0.83	0.83	0.82	0.81	0.82
Total WM	0.95	0.95	0.95	0.94	0.94	0.95	0.96	0.96	0.96
Total cerebellum	0.90	0.90	0.90	0.90	0.90	0.90	0.90	0.90	0.90
Cerebellar WM	0.87	0.87	0.87	0.89	0.89	0.88	0.86	0.85	0.87
Cerebellar GM	0.92	0.92	0.92	0.90	0.90	0.91	0.94	0.94	0.93
Cortical volume analysis									
Frontal									
Superior frontal gyrus, left	0.87	0.87	0.88	0.88	0.88	0.89	0.86	0.86	0.87
Superior frontal gyrus, right	0.87	0.87	0.88	0.86	0.86	0.86	0.89	0.88	0.89
Rostral middle frontal, left	0.85	0.85	0.85	0.86	0.86	0.85	0.84	0.84	0.85
Rostral middle frontal, right	0.83	0.83	0.84	0.89	0.89	0.89	0.78	0.78	0.79
Caudal middle frontal, left	0.80	0.79	0.81	0.78	0.77	0.80	0.82	0.82	0.82
Caudal middle frontal, right	0.78	0.79	0.77	0.73	0.73	0.73	0.85	0.86	0.82
Pars opercularis, left	0.81	0.81	0.82	0.82	0.81	0.83	0.81	0.81	0.81
Pars opercularis, right	0.77	0.78	0.75	0.81	0.81	0.80	0.74	0.75	0.71
Pars triangularis, left	0.82	0.82	0.81	0.83	0.83	0.83	0.81	0.81	0.80
Pars triangularis, right	0.82	0.82	0.82	0.80	0.80	0.79	0.84	0.84	0.85
Pars orbitalis, left	0.76	0.76	0.76	0.81	0.81	0.81	0.72	0.73	0.72
Pars orbitalis, right	0.77	0.77	0.75	0.83	0.84	0.82	0.72	0.73	0.70
Lateral orbitofrontal, left	0.85	0.84	0.86	0.82	0.82	0.82	0.89	0.88	0.90
Lateral orbitofrontal, right	0.84	0.83	0.84	0.79	0.79	0.79	0.89	0.89	0.90
Medial orbitofrontal, left	0.76	0.76	0.77	0.77	0.76	0.78	0.76	0.76	0.77
Medial orbitofrontal, right	0.78	0.78	0.79	0.78	0.78	0.77	0.80	0.79	0.81
Precentral, left	0.84	0.83	0.86	0.83	0.82	0.85	0.85	0.84	0.87
Precentral, right	0.84	0.85	0.82	0.84	0.85	0.82	0.84	0.86	0.82
Paracentral, left	0.78	0.77	0.79	0.78	0.76	0.81	0.79	0.78	0.80
Paracentral, right	0.77	0.77	0.77	0.80	0.79	0.83	0.75	0.76	0.73
Frontal pole, left	0.65	0.65	0.65	0.76	0.75	0.78	0.58	0.59	0.57
Frontal pole, right	0.66	0.67	0.66	0.78	0.77	0.80	0.59	0.60	0.58
Rostral anterior cingulate, left	0.78	0.78	0.79	0.76	0.76	0.75	0.82	0.81	0.83
Rostral anterior cingulate, right	0.76	0.76	0.75	0.75	0.76	0.73	0.77	0.77	0.78
Caudal anterior cingulate, left	0.72	0.72	0.73	0.75	0.75	0.73	0.71	0.70	0.74
Caudal anterior cingulate, right	0.74	0.73	0.76	0.80	0.78	0.82	0.71	0.70	0.72
Parietal									
Superior parietal, left	0.83	0.82	0.85	0.82	0.80	0.85	0.84	0.84	0.86
Superior parietal, right	0.81	0.80	0.83	0.79	0.78	0.82	0.84	0.84	0.84
Inferior parietal, left	0.82	0.81	0.84	0.83	0.82	0.84	0.82	0.80	0.84
Inferior parietal, right	0.83	0.82	0.84	0.85	0.85	0.86	0.81	0.80	0.82
Supramarginal, left	0.81	0.81	0.83	0.83	0.82	0.85	0.80	0.79	0.81
Supramarginal, right	0.82	0.83	0.81	0.85	0.86	0.85	0.80	0.81	0.79
Postcentral, left	0.81	0.80	0.83	0.82	0.81	0.84	0.80	0.79	0.82
Postcentral, right	0.79	0.79	0.77	0.79	0.80	0.78	0.78	0.79	0.77
Precuneus, left	0.84	0.84	0.85	0.81	0.80	0.83	0.89	0.88	0.89
Precuneus, right	0.83	0.83	0.84	0.80	0.79	0.81	0.87	0.87	0.88
Posterior cingulate, left	0.79	0.78	0.80	0.77	0.76	0.79	0.80	0.80	0.82
Posterior cingulate, right	0.78	0.77	0.78	0.74	0.75	0.74	0.82	0.81	0.83
Isthmus cingulate, left	0.79	0.78	0.79	0.83	0.83	0.83	0.75	0.75	0.76
Isthmus cingulate, right	0.76	0.75	0.76	0.82	0.82	0.82	0.70	0.70	0.72
Temporal									
Superior temporal, left	0.82	0.82	0.82	0.80	0.80	0.80	0.85	0.84	0.85
Superior temporal, right	0.85	0.85	0.86	0.81	0.82	0.81	0.90	0.89	0.91
Middle temporal, left	0.77	0.77	0.78	0.71	0.72	0.70	0.85	0.84	0.87
Middle temporal, right	0.81	0.81	0.82	0.77	0.78	0.77	0.86	0.84	0.88
Inferior temporal, left	0.72	0.72	0.71	0.70	0.71	0.68	0.74	0.74	0.75
Inferior temporal, right	0.74	0.73	0.76	0.72	0.71	0.74	0.77	0.76	0.79
Banks of the sts, left	0.68	0.68	0.67	0.68	0.70	0.66	0.68	0.67	0.70
Banks of the sts, right	0.71	0.69	0.73	0.71	0.69	0.75	0.71	0.71	0.72
Fusiform, left	0.73	0.74	0.73	0.76	0.75	0.77	0.72	0.72	0.71
Fusiform, right	0.76	0.75	0.77	0.79	0.78	0.81	0.73	0.73	0.74
Transverse temporal, left	0.77	0.77	0.77	0.73	0.73	0.73	0.81	0.81	0.82
Transverse temporal, right	0.76	0.75	0.77	0.77	0.77	0.77	0.76	0.75	0.77
Entorhinal, left	0.63	0.62	0.64	0.67	0.66	0.70	0.60	0.60	0.60
Entorhinal, right	0.62	0.62	0.62	0.56	0.55	0.56	0.74	0.75	0.71
Temporal pole, left	0.66	0.66	0.67	0.63	0.62	0.63	0.72	0.72	0.73
Temporal pole, right	0.64	0.63	0.66	0.67	0.67	0.67	0.62	0.60	0.65
Parahippocampal, left	0.77	0.76	0.78	0.83	0.83	0.84	0.72	0.71	0.73
Parahippocampal, right	0.76	0.75	0.76	0.83	0.82	0.84	0.70	0.70	0.71
Occipital									
Lateral occipital, left	0.79	0.79	0.81	0.77	0.76	0.77	0.83	0.82	0.85
Lateral occipital, right	0.78	0.78	0.78	0.69	0.69	0.69	0.89	0.89	0.90
Lingual, left	0.79	0.79	0.80	0.75	0.74	0.75	0.85	0.85	0.86
Lingual, right	0.80	0.80	0.81	0.79	0.79	0.79	0.81	0.81	0.83
Cuneus, left	0.74	0.73	0.76	0.71	0.70	0.73	0.78	0.77	0.80
Cuneus, right	0.75	0.75	0.75	0.77	0.76	0.77	0.73	0.73	0.73
Pericalcarine, left	0.72	0.71	0.74	0.72	0.70	0.76	0.73	0.72	0.73
Pericalcarine, right	0.74	0.73	0.75	0.67	0.65	0.69	0.84	0.83	0.84
Insular cortex, left	0.84	0.84	0.84	0.81	0.81	0.80	0.88	0.87	0.89
Insular cortex, right	0.85	0.85	0.85	0.85	0.85	0.85	0.85	0.85	0.86
Subcortical volume analysis									
Hippocampus, left	0.85	0.85	0.86	0.86	0.86	0.86	0.85	0.84	0.86
Hippocampus, right	0.86	0.86	0.85	0.84	0.85	0.83	0.88	0.88	0.88
Thalamus, left	0.88	0.88	0.88	0.88	0.88	0.88	0.88	0.88	0.89
Thalamus, right	0.91	0.91	0.91	0.91	0.91	0.91	0.90	0.90	0.90
Putamen, left	0.84	0.84	0.84	0.91	0.91	0.91	0.79	0.79	0.79
Putamen, right	0.86	0.86	0.87	0.94	0.94	0.94	0.80	0.79	0.81
Caudate, left	0.88	0.88	0.89	0.92	0.92	0.93	0.85	0.84	0.86
Caudate, right	0.88	0.88	0.88	0.87	0.87	0.87	0.89	0.89	0.89
Pallidum, left	0.76	0.77	0.75	0.75	0.76	0.72	0.78	0.78	0.78
Pallidum, right	0.79	0.79	0.78	0.78	0.79	0.77	0.80	0.80	0.79
Accumbens, left	0.71	0.70	0.73	0.69	0.68	0.70	0.75	0.74	0.77
Accumbens, right	0.64	0.64	0.66	0.77	0.76	0.78	0.56	0.56	0.57

*GM* gray matter, *WM* white matter.

## Discussion

In pediatric populations, accurate brain segmentations of MR imaging can help find a diagnostic biomarker for a specific neurological disease, define a clinical course, or identify the underlying developmental patho-mechanism of various neurodevelopmental disorders^24^. However, accurate segmentation of pediatric brain MR imaging is challenging due to the reduced tissue contrast, increased noise, several partial volume effects, and ongoing white matter myelination^25,26^. This study aimed to demonstrate the performance of the DLS method for brain segmentation of MRI in a pediatric population aged under 11 years.

To investigate the accuracy of the DLS method in measuring regional brain volume, the volume of whole brain (Table 1 and Fig. 1), parcellated cortical volumes (Table 2), and segmented subcortical volumes (Table 3 and Fig. 2) were measured by a DLS method in healthy controls and compared to previously measured volumes using Freesurfer with a manual editing method.

Importantly, using the DLS method, the volumes of the total brain, total cerebral gray matter, cortical gray matter, and total white matter, were consistent with those measured by Freesurfer with manual editing (Table 1 and Fig. 1). In particular, the DLS methods can successfully delineate the gray and white matter and parcellate the total cortical gray matter (Table 1 and Fig. 1) as the freesurfer did (Table 2) representing the good performance of DLS method for cortical volume analysis.

The volume of only seven area including right caudal, middle frontal, right frontal pole, left inferior parietal, left fusiform, left lateral occipital, right insular, and the left parahippocampal cortex, measured by the DLS method, were discordant with that measured by Freesurfer with manual correction. After a subgroup analysis, the differences were resolved in the Age > 2 years group.

Since CNN was first introduced in 1989^27^, great interest in CNN’s ability of the neonate and infant brain segmentation were gained through two large-scale competitions using standardized open data sets: the Neonatal Brains Segmentation Challenge and the 2017 iSeg 6-month Infant Brain Magnetic Resonance Imaging Segmentation Challenge. They concluded that the CNN approach, using deep-learning methods, could solve the neonate and 6-month-old brain tissue segmentation with a respectable dice similarity coefficient of 72.5–73.5%^6,28,29^.

Recently, the UNC/UMN Baby Connectome Project (https://iseg2017.web.unc.edu/baby-connectome-project/) is in the process of identifying brain and behavioral development in typically developing infants across the first 5 years of life by analyzing structural segmentation and functional connectivity^30^. These data suggest that CNN can be applied to the segmentation of young child brains and that it is particularly effective in whole brain and cortical gray matter volume analysis. In addition, currently released deep learning-based, infant-dedicated cortical surface reconstruction pipeline, iBEAT V2.0 were successfully processed various imaging protocols/scanners^31^.

However, there were volume differences between Freesurfer with manual editing, and the new DLS method in the cerebellum and subcortical gray matter, including both thalami and the right putamen. Regard to subgroup analysis, the volume differences were evident mainly in patients of age under two and also dissolved in older age groups upper two as cortical parcellated volume analysis. The Supplementary Fig. 1 provide several illustrative examples, highlighting enhancements in achieving consistent and accurate segmentation in specific brain areas using deep learning segmentation (DLS) methods. Notably, the examples include incorrect segmentation from subcortical gray matter (GM) to white matter (WM) (Supplementary Fig. 1A), a noisy boundary in cerebellar segmentation (Supplementary Fig. 1B), and smaller segmented volumes in the putamen and pallidum areas (Supplementary Fig. 1C and D), when measured by Freesurfer with manual editing. These findings imply that the DLS approach could be instrumental in addressing the challenges of precise segmentation of complex structures, which is a limitation observed with Freesurfer.

Traditionally, atlas-based methods^32–34^, which match intensity information between an atlas and target images, and pattern recognition methods^35,36^, which classify tissues based on a set of local intensity features, are the classical approaches to automated segmentation^11^. Unfortunately, these methods have been shown to provide inaccurate segmentation for pediatric brain^37,38^ due to an inappropriate template, customized to adult brains with low intensity contrasts and high shape variability of each regional structure, including the thalamus, hippocampus, parahippocampal areas, and insular cortex. For these reasons, volume differences measured between these two methods can be observed in these areas. Additionally, small sample sizes with large dispersions also contribute to the volume differences measured between the two methods.

In investigating the ability to identify brain morphometric abnormalities in patients with a *SCN1A* mutation, we compared the volumes between patients and healthy controls using the DLS method (Table 4). In whole brain analysis, the volumes of total brain, total gray matter, cortical gray matter, subcortical gray matter, and total white matter were significantly decreased in patients with a *SCN1A* mutation and related epilepsy, compared to that of healthy control and these results were consistent with our previous study^22^. In addition to the cortical and subcortical areas which showed reduced volume in patients using Freesurfer software with manual correction^22^, the both thalamus, right pallidum, right lateral orbitofrontal, right paracentral, right inferior parietal, left Banks of the STS, left parahippocampal, and right insular cortex were significantly smaller in patients with *SCN1A* mutation related epilepsy compared to those of healthy controls in this study.

The banks of the STS, and parahippocampal cortex are subnetworks of default mode network (DMN) and the thalamus and insular cortex are correlated areas with DMN in studies with resting state functional MRIs^39,40^. These structural alterations of the DMN and DMN-associated areas, in patients with a *SCN1A* mutation, is consistent with the results of other studies^22,41^.

For cortical parcellation, FreeSurfer generates a white matter (WM) surface and pial surface for each hemisphere. The WM surface is generated from a segmentation mask and a copy of the WM surface is deformed towards the cerebrospinal fluid (CSF)/Gray matter (GM) boundary, to eventually form a pial surface. The cortical surface is mapped to a spherical atlas and the probabilities for each cortical region are calculated with Bayesian estimations. As the parcellation pipeline includes intensive computation such as registration and surface reconstruction, the whole processing time is around 7 h while it depends on the computation environment.

Since DeepBrain utilizes the fully trained deep-learning model for the parcellation pipeline that replaces manually designed algorithms such as cortical surface generation, the computation time can be significantly reduced. The total computation time for whole brain parcellation was less than 1 min, which is incomparably faster than traditional methods, such as FreeSurfer.

Despite several limitations, namely lack of validation using standardized open datasets or other datasets from multiple resources with dice coefficient, and a small number of patients, the deep-learning-based method with CNN could provide a key solution for accurate pediatric brain segmentation. Importantly, accurate and quick segmentation by CNN may identify the normal trajectories of developing brains, leading ultimately to the early detection and treatment of various neurodevelopmental diseases.

In a pediatric population, this new, fully automated DLS method is compatible with the classic, volumetric analysis with Freesurfer software and manual correction, and it can also well detect segmental brain volume change in children with a *SCN1A* mutation. Further validation using larger population data may confirm that this fully automated DLS method is a good and easy tool for accurate brain segmentations in pediatric populations.

## Methods

### Subjects

The 21 patients of epilepsy with a *SCN1A* mutation, who were under 11 years of age, and the 42 healthy controls, who had participated in our previous study, were reinvestigated by the DLS method^22^. Patients of epilepsy with a *SCN1A* mutation were recruited from the pediatric neurology clinics of three medical centers in Korea: Asan Medical Center, Samsung Medical Center, and Seoul National University Children’s Hospital. We included patients who satisfied the following inclusion criteria: (i) epilepsy diagnosed by a pediatric neurologist; (ii) a genetically confirmed *SCN1A* mutation and (iii) a normal brain MRI. For each patient, two healthy control subjects matched in age and sex and without alleged neurologic deficits, were recruited.

### MRI acquisition

MRI scans were obtained on a Philips Achieva 3.0 T scanner (Philips Healthcare, Eindhoven, The Netherlands) (n = 114) and Siemens MAGNETOM Verio 3.0 T scanner (Siemens AG, Erlangen, Germany) (n = 6). Three-dimensional whole brain T1 sequence imaging was acquired with the following image parameters: echo time (TE) = 4.6 ms, repetition time (TR) = 9.8 ms, flip angle (FA) = 8.08, field of view (FOV) = 224 × 224 mm, matrix = 256 × 256, slice thickness = 1 mm, sagittal images of the entire brain with in-plane resolution 1.0 mm × 1.0 mm or TE = 5.1 ms, TR = 25 ms, FA = 30, FOV = 220 × 220 mm, matrix = 512 × 512, slice thickness = 1 mm, sagittal images of the entire brain with in-plane resolution 1.0 mm × 1.0 mm on a Philips 3.0 T Achieva scanner. On the MAGNETOM Verio scanner, images were obtained with TE = 1.9 ms, TR = 1500 ms, FA = 9.0, FOV = 220 × 220 mm, matrix = 256 × 256, slice thickness = 1 mm, sagittal images of the entire brain with in-plane resolution 1.0 mm × 1.0 mm. Prior to data processing, all raw T1 sequencing images were visually inspected for common MR T1 weighted imaging artifacts.

### Image analysis

We analyzed T1-weighted MR images using two automated segmentation software, VUNO Med-DeepBrain (version 1.0.1, VUNO Inc., Seoul, South Korea)^42,43^ and FreeSurfer (version 5.3.0, https://surfer.nmr.mgh.harvard.edu)^42,43^. FreeSurfer is a publicly available software for brain analysis which provides automated brain segmentation and cortical parcellation. It uses a probabilistic atlas generated from manually segmented brain MR images to train a Bayesian segmentation algorithm.

VUNO Med-DeepBrain is based on DLS system, unlike the atlas-based segmentation methods used in FreeSurfer and NeuroQuant. The DLS system provides quantitative information, which includes the volume of 104 regions and cortical thickness of 68 cortical regions (34 for each hemisphere) from the T1-weighted brain MRI, and white matter hyperintensity (WMH) regions from the T2-weighted brain MRI. The DLS system is designed using in-house segmentation model with convolutional neural networks (CNNs) with dilated convolution layers instead of max-pooling to minimize feature loss of small brain regions during spatial dimension reduction. The model was originally trained with adult MR images, but we fine-tuned the model with additional pediatric brain images obtained from OpenfMRI dataset^44,45^. Fine-tuning dataset consists of 249 images (female: 156, male: 143, age: 5.01–19.22, σ = 4.33). The input image comprised a conformed T1-weighted brain MRI and the outputs are segmented brain regions mask and associated volumes of 104 regions in total. The CNNs was trained using ADAM optimizer and generalized dice loss function. The DLS system also includes a 3D CNN model that provides the intracranial volume segmentation, which was used to normalize the volume measurements for statistical analysis.

The total processing time including image preprocessing and normalized volume retrieval was about 1 min on the minimum computational requirement setting (CPU: 16 GB, GPU: RTX2080Ti 11 GB). Although the model was trained with both 3 T and 1.5 T images, exploiting 3 T MR images was recommended to acquire more accurate segmentation.

### Statistical analysis

We compared the absolute volume differences of healthy controls measured by two methods using the paired t-test. For multiple comparisons, a Bonferroni correction was applied, and *p* < *0.001* was considered significant for the whole brain, the 10 subcortical, and 34 cortical volumes.

The adjusted volumes ($Vo{lume}_{adj}$) were used for comparisons between the control and patient groups by the DLS method, in order to adjust the total (ICV). $Vo{lume}_{adj}$ signifies the linearly adjusted volume calculated as $Volume-\beta (ICV-IC{V}_{mean})$ where $IC{V}_{mean}$ is the mean ICV of the each group and the parameter was fixed to minimize the covariance of the $Vo{lume}_{adj}$ and ICVs in each group^46^.

We fitted linear models with the interceptor term and the group indicator (healthy control vs. *SCN1A* patients) as covariates and used generalized least squares to estimate regression coefficients. The errors were allowed to be correlated with unconstrained parameters. After the Bonferroni correction for multiple comparison, a *p* < *0.001* was considered significant for the whole brain, the 10 subcortical, and 34 cortical volumes.

To determine the spatial overlap of the structures, we conducted Dice score coefficient (DSC), precision, and recall analysis between manual and automated segmentation methods. Dice Score can be defined as twice the total overlapping area of the predicted mask and the ground truth divided by the sum of the total number of tumor pixels (i.e. pixel value 1 [foreground]) in both the predicted mask and the ground truth). The value of DSC ranges from 0, indicating no spatial overlap between structures, to 1, indicating complete overlap^47^.

Other metrics used require pixel-wise evaluation of the ground truth and the predicted masks. True positive (TP) can be defined as the total number of positive pixels (belonging to the tumor) in the ground truth which are correctly predicted, True negative (TN) is the total number of negative pixels (belonging to the background) in the ground truth which are correctly predicted negative, False positive (FP) is the total number of negative pixels which are falsely predicted as positive pixels. Precision can be defined as the ratio of the total number of pixels predicted as positive to the total number of actual foregrounds. Recall is the ratio of the total number of correctly predicted foregrounds to the total number of actual foregrounds.

### Ethical approval and consent of participate

The study protocol was reviewed and approved by the Institutional Review Board of the University of Ulsan College of Medicine (No. 2014-0405), and informed consent was waived because of the retrospective nature of the study. The Study was conducted in accordance with the Declaration of Helsinki.

### Supplementary Information

Supplementary Information.

## Data Availability

The data that support the findings of this study are available on request from the corresponding author. The data are not publicly available due to privacy or ethical restrictions.
