# Supplementary material for: Deep learning-based, fully automated, pediatric brain segmentation
Source: Sci Rep. 2024 Feb 22;14:4344. doi: 10.1038/s41598-024-54663-z (PMC10881508; doi:10.1038/s41598-024-54663-z)

**Supplementary table 1. Cortical and subcortical parcellated volumes by deep-learning based segmentation (DLS) method in healthy and *SCN1A* patients**

| **Cortical volume analysis** | | | | |
| --- | --- | --- | --- | --- |
|  | **Left** | | **Right** | |
|  | **Healthy control**  **(n = 42)** | ***SCN1A* patients**  **(n=21)** | **Healthy control**  **(n = 42)** | ***SCN1A* patients**  **(n=21)** |
| **Frontal** | | | | |
| **Superior frontal gyrus** | 25.6 | 23.5 | 24.2 | 22.0 |
| **Rostral middle frontal** | 18.8 | 16.9 | 19.4 | 17.1 |
| **Caudal middle frontal** | 7.2 | 6.9 | 7.5 | 6.8 |
| **Pars opercularis** | 5.5 | 4.8 | 4.6 | 4.3 |
| **Pars triangularis** | 4.6 | 4.1 | 5.3 | 4.9 |
| **Pars orbitalis** | 2.8 | 2.5 | 3.0 | 2.7 |
| **Lateral orbitofrontal** | 9.2 | 8.1 | 8.1 | 7.2 |
| **Medial orbitofrontal** | 6.2 | 6.0 | 6.4 | 6.1 |
| **Precentral** | 14.5 | 13.4 | 14.4 | 13.3 |
| **Paracentral** | 4.1 | 4.1 | 4.1 | 3.8 |
| **Frontal pole** | 1.4 | 1.4 | 1.9 | 1.7 |
| **Rostral anterior cingulate** | 2.9 | 2.4 | 2.2 | 1.9 |
| **Caudal anterior cingulate** | 1.8 | 1.7 | 2.3 | 2.2 |
| **Parietal** | | | | |
| **Superior parietal** | 17.9 | 16.3 | 16.6 | 16.1 |
| **Inferior parietal** | 16.3 | 14.5 | 21.8 | 18.1 |
| **Supramarginal** | 14.4 | 13.8 | 11.6 | 10.8 |
| **Postcentral** | 11.4 | 11.1 | 11.1 | 10.3 |
| **Precuneus** | 12.6 | 11.3 | 13.4 | 11.9 |
| **Posterior cingulate** | 3.9 | 3.5 | 4.0 | 3.6 |
| **Isthmus cingulate** | 3.4 | 3.1 | 3.3 | 2.9 |
| **Temporal** | | | | |
| **Superior temporal** | 14.1 | 13.0 | 13.3 | 12.4 |
| **Middle temporal** | 12.4 | 11.0 | 15.3 | 13.2 |
| **Inferior temporal** | 12.8 | 11.9 | 11.2 | 11.5 |
| **Banks of the STS** | 3.0 | 2.5 | 2.8 | 2.4 |
| **Fusiform** | 12.4 | 11.3 | 11.0 | 10.1 |
| **Transverse temporal** | 1.5 | 1.6 | 1.4 | 1.3 |
| **Entorhinal** | 1.8 | 1.6 | 1.5 | 1.5 |
| **Temporal pole** | 2.6 | 2.4 | 2.4 | 2.3 |
| **Parahippocampal** | 2.0 | 1.7 | 2.0 | 1.9 |
| **Occipital** | | | | |
| **Lateral occipital** | 14.6 | 12.8 | 15.4 | 14.0 |
| **Lingual** | 7.9 | 7.4 | 8.0 | 7.4 |
| **Cuneus** | 3.4 | 3.2 | 3.8 | 3.5 |
| **Pericalcarine** | 2.5 | 2.4 | 2.6 | 2.4 |
| **Insular cortex** | 7.0 | 6.6 | 7.2 | 6.7 |
| **Subcortical volume analysis** | | | | |
|  | **Left** | | **Right** | |
|  | **Healthy control**  **(n = 42)** | ***SCN1A* patients**  **(n=21)** | **Healthy control**  **(n = 42)** | ***SCN1A* patients**  **(n=21)** |
| **Thalamus** | 7.2 | 6.9 | 7.3 | 6.8 |
| **Putamen** | 5.2 | 4.6 | 5.0 | 4.5 |
| **Caudate** | 3.5 | 3.2 | 3.6 | 3.2 |
| **Pallidum** | 1.9 | 1.8 | 1.9 | 1.7 |
| **Accumbens** | 0.7 | 0.6 | 0.6 | 0.6 |

Volume is expressed in mL

**Supplementary table 2. Whole brain, Cortical and subcortical parcellated volumes by deep-learning based segmentation (DLS) method in healthy and *SCN1A* patients according to sex**

| **Whole brain volume analysis** | | | | |
| --- | --- | --- | --- | --- |
|  | **Healthy control (n = 42)** | | **Patients (n = 21)** | |
|  | **Male**  **(n = 14)** | **Female**  **(n = 28)** | **Male**  **(n = 7)** | **Female**  **(n = 14)** |
| **Total brain** | 1167.5 | 1106.8 | 1093.4 | 1075.3 |
| **Total GM** | 626.8 | 602.1 | 581.4 | 568.9 |
| **Cortical GM** | 581.3 | 558.8 | 539.6 | 527.1 |
| **Subcortical GM** | 45.5 | 43.4 | 41.8 | 41.7 |
| **Total WM** | 376.7 | 349.9 | 350.8 | 348.2 |
| **Total cerebellum** | 132.5 | 126.5 | 130.4 | 128.3 |
| **Cerebellar WM** | 22.2 | 20.7 | 22.7 | 21.2 |
| **Cerebellar GM** | 110.2 | 105.8 | 107.7 | 107.1 |
| **Cortical volume analysis** | | | | |
| **Frontal** | | | | |
| **Superior frontal gyrus, Left** | 26.5 | 25.1 | 24.5 | 24.1 |
| **Superior frontal gyrus, Right** | 25.0 | 23.8 | 22.2 | 23.0 |
| **Rostral middle frontal, Left** | 19.0 | 18.6 | 16.6 | 17.9 |
| **Rostral middle frontal, Right** | 20.2 | 19.0 | 17.3 | 18.3 |
| **Caudal middle frontal, Left** | 7.4 | 7.1 | 7.2 | 7.2 |
| **Caudal middle frontal, Right** | 7.4 | 7.6 | 6.9 | 7.1 |
| **Pars opercularis, Left** | 5.8 | 5.4 | 5.0 | 5.2 |
| **Pars opercularis, Right** | 4.6 | 4.6 | 4.0 | 4.6 |
| **Pars triangularis, Left** | 4.9 | 4.5 | 4.5 | 4.1 |
| **Pars triangularis, Right** | 5.8 | 5.1 | 5.1 | 5.0 |
| **Pars orbitalis, Left** | 2.9 | 2.8 | 2.4 | 2.7 |
| **Pars orbitalis, Right** | 2.9 | 3.0 | 2.7 | 2.8 |
| **Lateral orbitofrontal, Left** | 9.4 | 9.2 | 8.7 | 8.3 |
| **Lateral orbitofrontal, Right** | 8.3 | 8.0 | 7.8 | 7.2 |
| **Medial orbitofrontal, Left** | 6.3 | 6.2 | 6.3 | 6.2 |
| **Medial orbitofrontal, Right** | 6.6 | 6.3 | 6.5 | 6.1 |
| **Precentral, Left** | 14.8 | 14.3 | 13.4 | 14.0 |
| **Precentral, Right** | 14.6 | 14.3 | 13.2 | 13.9 |
| **Paracentral, Left** | 4.1 | 4.1 | 3.9 | 4.2 |
| **Paracentral, Right** | 4.3 | 4.1 | 3.7 | 3.9 |
| **Frontal pole, Left** | 1.4 | 1.4 | 1.2 | 1.4 |
| **Frontal pole, Right** | 1.9 | 1.9 | 1.6 | 1.7 |
| **Rostral anterior cingulate, Left** | 3.1 | 2.9 | 2.5 | 2.7 |
| **Rostral anterior cingulate, Right** | 2.3 | 2.2 | 2.1 | 1.9 |
| **Caudal anterior cingulate, Left** | 1.8 | 1.8 | 1.5 | 1.9 |
| **Caudal anterior cingulate, Right** | 2.5 | 2.2 | 2.4 | 2.2 |
| **Parietal** | | | | |
| **Superior parietal, Left** | 18.1 | 17.7 | 16.3 | 16.5 |
| **Superior parietal, Right** | 16.7 | 16.5 | 16.5 | 15.9 |
| **Inferior parietal, Left** | 16.8 | 16.1 | 14.6 | 14.6 |
| **Inferior parietal, Right** | 22.3 | 21.6 | 19.5 | 18.1 |
| **Supramarginal, Left** | 14.2 | 14.5 | 14.6 | 14.0 |
| **Supramarginal, Right** | 11.9 | 11.5 | 11.4 | 11.1 |
| **Postcentral, Left** | 12.0 | 11.2 | 11.5 | 11.3 |
| **Postcentral, Right** | 11.9 | 10.7 | 10.3 | 10.8 |
| **Precuneus, Left** | 12.9 | 12.4 | 11.8 | 11.1 |
| **Precuneus, Right** | 13.8 | 13.2 | 12.7 | 11.6 |
| **Posterior cingulate, Left** | 4.0 | 3.8 | 3.4 | 3.7 |
| **Posterior cingulate, Right** | 4.1 | 4.0 | 3.7 | 3.9 |
| **Isthmus cingulate, Left** | 3.5 | 3.3 | 3.2 | 3.0 |
| **Isthmus cingulate, Right** | 3.3 | 3.3 | 3.1 | 2.9 |
| **Temporal** | | | | |
| **Superior temporal, Left** | 14.1 | 14.1 | 14.4 | 13.1 |
| **Superior temporal, Right** | 13.3 | 13.3 | 13.9 | 12.5 |
| **Middle temporal, Left** | 13.0 | 12.1 | 10.8 | 11.8 |
| **Middle temporal, Right** | 15.7 | 15.0 | 14.2 | 13.7 |
| **Inferior temporal, Left** | 13.4 | 12.6 | 13.1 | 12.0 |
| **Inferior temporal, Right** | 11.2 | 11.2 | 12.4 | 11.4 |
| **Banks of the STS, Left** | 3.1 | 3.0 | 3.0 | 2.4 |
| **Banks of the STS, Right** | 2.9 | 2.8 | 2.4 | 2.5 |
| **Fusiform, Left** | 12.8 | 12.2 | 12.3 | 11.3 |
| **Fusiform, Right** | 11.6 | 10.7 | 11.3 | 10.1 |
| **Transverse temporal, Left** | 1.7 | 1.5 | 1.7 | 1.5 |
| **Transverse temporal, Right** | 1.5 | 1.3 | 1.4 | 1.3 |
| **Entorhinal, Left** | 1.9 | 1.8 | 1.9 | 1.6 |
| **Entorhinal, Right** | 1.6 | 1.4 | 1.6 | 1.6 |
| **Temporal pole, Left** | 2.7 | 2.5 | 2.6 | 2.4 |
| **Temporal pole, Right** | 2.5 | 2.4 | 2.6 | 2.3 |
| **Parahippocampal, Left** | 2.1 | 2.0 | 1.8 | 1.8 |
| **Parahippocampal, Right** | 2.1 | 2.0 | 1.9 | 1.9 |
| **Occipital** | | | | |
| **Lateral occipital, Left** | 15.5 | 14.1 | 14.5 | 12.3 |
| **Lateral occipital, Right** | 16.1 | 15.1 | 14.7 | 13.9 |
| **Lingual, Left** | 8.0 | 7.9 | 7.7 | 7.3 |
| **Lingual, Right** | 7.8 | 8.1 | 7.8 | 7.3 |
| **Cuneus, Left** | 3.7 | 3.3 | 3.4 | 3.2 |
| **Cuneus, Right** | 4.2 | 3.7 | 3.8 | 3.5 |
| **Pericalcarine, Left** | 2.3 | 2.7 | 2.3 | 2.4 |
| **Pericalcarine, Right** | 2.6 | 2.6 | 2.4 | 2.4 |
| **Insular cortex, Left** | 7.2 | 6.9 | 6.9 | 6.7 |
| **Insular cortex, Right** | 7.6 | 7.1 | 7.0 | 6.8 |
| **Subcortical volume analysis** | | | | |
| **Thalamus, Left** | 7.5 | 7.1 | 7.3 | 6.9 |
| **Thalamus, Right** | 7.6 | 7.2 | 7.2 | 6.9 |
| **Putamen, Left** | 5.5 | 5.1 | 4.7 | 4.8 |
| **Putamen, Right** | 5.3 | 4.9 | 4.6 | 4.6 |
| **Caudate, Left** | 3.6 | 3.5 | 3.2 | 3.4 |
| **Caudate, Right** | 3.7 | 3.5 | 3.1 | 3.4 |
| **Pallidum, Left** | 1.9 | 1.9 | 1.9 | 1.8 |
| **Pallidum, Right** | 1.9 | 1.8 | 1.8 | 1.7 |
| **Accumbens, Left** | 0.7 | 0.7 | 0.6 | 0.6 |
| **Accumbens, Right** | 0.6 | 0.6 | 0.6 | 0.6 |

Volume is expressed in mL

**Supplementary Figure 1. Segmentation Discrepancies of several areas in DeepBrain and FreeSurfer Methods.** (A) Subcortical Gray Matter to White Matter: Demonstrates incorrect segmentation, highlighting the challenges in delineating these regions accurately. (B) Cerebellar Segmentation: Illustrates a noisy boundary, indicating difficulties in consistent segmentation of this area. (C) Putamen and (D) Pallidum areas: Shows smaller segmented regions, reflecting potential underestimation of these areas.


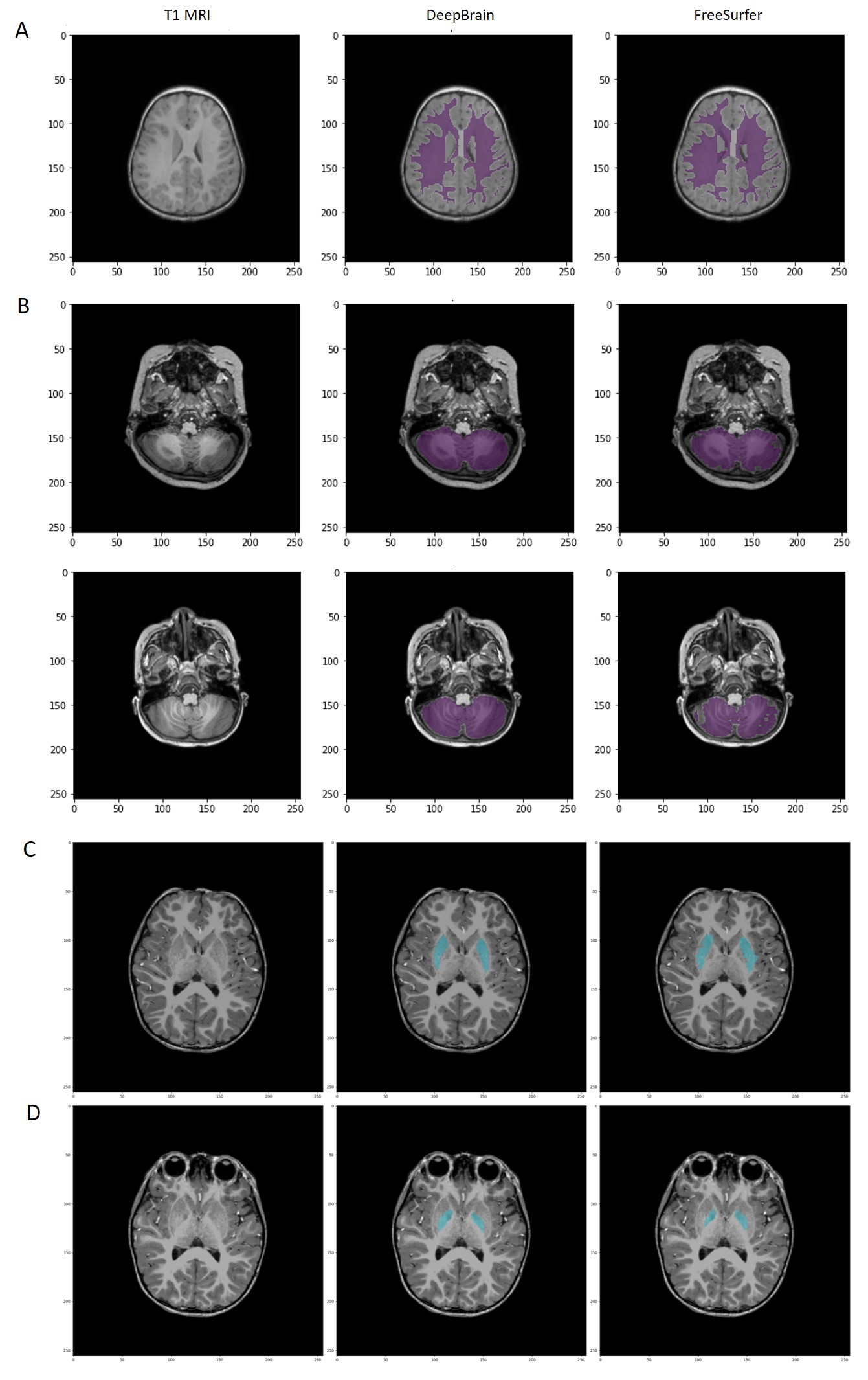

Supplement: Supplementary file 1 — Supplementary Information. [file 41598_2024_54663_MOESM1_ESM.docx]
